# Prevalence, attitude, misconception, and predictors of electronic cigarette among King Khalid University students, Abha, Aseer region, Saudi Arabia

**DOI:** 10.1097/MD.0000000000042229

**Published:** 2025-04-18

**Authors:** Nasser G. Alqahtani, Amira Mohamed Taha, Reem Hamad S. Aldosari, Lama Gasem Asiri, Lujain Y. AlKasi, Shatha A. Almasswary, Alshaima Alassim, Aljohrah M. Al Hunaif, Shuruq Abdullah M. Alqahtani, Miad A. Abu Mughaedh, Amr Ehab El-Qushayr

**Affiliations:** a Department of Internal Medicine, Cardiology Section, College of Medicine, King Khalid University, Abha, Saudi Arabia; b Faculty of Medicine, Fayoum University, Fayoum, Egypt; c College of Medicine, King Khalid University, Abha, Saudi Arabia; d Faculty of Medicine, Minia University, Minia, Egypt.

**Keywords:** cross-sectional, e-cigarettes, electronic cigarettes, King Khaled

## Abstract

This research aims to explore the prevalence and perspectives on e-cigarette use at King Khalid University in Saudi Arabia. This is a cross-sectional study that was conducted during April and May 2023, recruiting students from the College of Medicine at King Khalid University, from Aseer region, Saudi Arabia. We included 534 participants, of those 19.3% had used electronic-cigarettes (e-cigarettes). The most reported reason for using e-cigarettes were reduce/quit conventional smoking because it is not a healthy habit (23%). A significantly lesser proportion of E. users rather than the users, think that e-cigarettes are a source of secondhand smoking, causing various diseases, burning tobacco as a source of power and harmful to pregnant women. Furthermore, the prevalence of the 2 groups was comparable when they were asked if e-cigarette is a nicotine delivery system and it contains addictive flavor. Female sex is inversely associated with e-cigarette smoking (odds ratio: 0.25 (0.15–0.42), *P* < .001). However, age, grade point average, and MBBS year were not associated with E. smoking. One fifth of the medical students at King Khaled University are E. users with a large proportion of males. The health care authorities in conjunction with the educational authorities should raise the awareness level towards e-cigarettes use in particular the reasons for usage as well as it is harmful hazards.

## 1. Introduction

Despite the global prevalence of tobacco smoking, with a significant user base exceeding 1.3 billion individuals, there is an emerging shift from traditional cigarettes towards electronic cigarettes (e-cigarettes).^[1,2]^ E-cigarettes have become a popular choice among individuals considering alternatives to traditional smoking worldwide.^[3,4]^ The fundamental design of an e-cigarette includes a battery, an atomizer for heating and a cartridge for storing a liquid.^[5]^ The liquid typically comprises propylene glycol, glycerol, and optionally nicotine, offering a less harmful substitute for tobacco smoke regarding cardiovascular hazards.^[6,7]^ The broad range of flavored e-liquids available in the e-cigarette market accommodates to a wide variety of preferences in order to quit traditional smoking.^[8]^ According to a 2014 estimate, there are around 7700 unique e-liquid flavors, which are likely to have expanded since then.^[9]^ Survey data from dedicated e-cigarette users showed a trend of beginning with tobacco-flavored e-cigarettes and gradually shifting to nontobacco flavors, particularly fruit, sweet, and dessert variants, as usage frequency and duration rise.^[10,11]^ Despite being regarded as less dangerous than traditional tobacco smoke, e-cigarette toxicity levels can vary greatly between brands.^[12]^ In September 2015, Saudi Arabia banned the sale of all e-cigarette. A few years later, e-cigarettes use was approved by the authorities with some restrictions in some places such as public transport, public places, and workspaces.

Emerging evidence indicated that e-cigarette use may be associated with mental health risks, contributing to a spectrum of cognitive and behavioral issues, such as learning difficulties, aggression, and depression.^[13,14]^ In addition, e-cigarette users are at an increased risk of physical harm, including thermal injuries from battery explosions,^[15]^ and cardiovascular complications like myocardial infarction.^[16]^ Furthermore, substances in e-cigarette vapor, notably acrolein, are implicated in enhancing neutrophil activity in the lungs, leading to possible lung tissue damage and broader health complications.^[17]^ The role of e-cigarettes in smoking cessation remains a subject of intense debate and study. A 2015 meta-analysis suggested that e-cigarettes might be more effective in aiding smokers to quit compared to a placebo.^[18]^ Compared to FDA-approved cessation aids or no aid at all, the use of e-cigarettes demonstrated similar rates of abstinence over a two-year period.^[19]^ The efficacy of e-cigarettes as a cessation tool continues to be a contentious topic, with the scientific community yet to reach a consensus. A recent trial that included participants who shifted from traditional smoking to either e-cigarettes or complete smoking cessation (control), indicated that there was no significant difference regarding the quality of life in the e-cigarettes group and the control group.^[20]^

In Saudi Arabia, a 2018 national survey revealed a smoking rate of 21.4%,^[21]^ with a higher prevalence among males. The prevalence of e-cigarette use among medical students in Saudi Arabia showed notable regional variations. Studies from 2019 and 2020 report rates ranging from 10.6% in Qassim^[22]^ to 27.7% in Jeddah,^[23]^ with a 2019 meta-analysis indicating an overall smoking prevalence of 17% among college students, higher in males than females.^[24]^ However, data regarding e-cigarette usage in the Aseer region is limited, which has led to the initiation of this study. This research aims to explore the prevalence and perspectives on e-cigarette use at King Khalid University in Saudi Arabia.

## 2. Method

### 2.1. Study design and setting

This is a cross-sectional study that was conducted during April and May 2023, recruiting students from the College of Medicine at King Khalid University, from Aseer region, Saudi Arabia.

### 2.2. Study population and sample size

The total number of medical students in King Khaled University, is approximately 1200 students. We used a non-probability sampling technique in the form of convenience sampling.

### 2.3. Inclusion and exclusion criteria

Medical students who use or do not use electronic cigarettes both males and females from all the levels participated based on their availability and willingness to participate in the study. We excluded participants who did not sign an informed consent, had incomplete data or participants from other colleges rather than college of medicine.

### 2.4. Data collocation and study questionnaire

To collect the data, we used a validated questionnaire that we uploaded to Google form and was distributed to all the medical students who use or do not use electronic cigarettes. The survey was already performed by Alshanberi et al, and was used in Umm Al-Qura University, Saudi Arabia since February 2020.^[25]^ Part 1 included the demographic characteristics of the students: their gender, age, educational levels, and grade point average (GPA). Part 2 included current smokers’ status and misconceptions regarding electronic smoking.

### 2.5. Ethical approval and consent

Before starting the study, its objectives were explained to the students, and informed consent was obtained. The ethical approval for this study was granted by the Research Ethics Board of the University of King Khalid with an approval number (ECM#2023-2109) in adherence to standards of ethical conduct for research on human subjects. Participants’ privacy and data confidentially were ensured across all phases of the project. Students were informed that the information collected was confidential and restricted in access, limited solely to the principal investigator and the statistician. It was explicitly conveyed that these data would not be utilized for any other publication. Students’ personal information will remain confidential and will not be made publicly available. Only the collected survey data will be used, and exclusively for the publication of this study.

### 2.6. Statistical analysis

We used SPSS version 24 to analyze our data. Chi-square test was used to detect the difference in e-cigarette knowledge between e-cigarette users and nonusers. Moreover, we used multivariable logistic regression analysis to determine predictors of e-cigarette use and reported the results into odds ratio and 95% confidence interval. If *P* value < .05, it was considered significant.

## 3. Results

We included 534 participants, of those 19.3% had used e-cigarettes (Table 1). Most participants were between 18 and 25 years old. The e-cigarette users had significantly higher prevalence of males reaching 75% compared to 43% of the non-users. Both groups had nearly an equal representation of all university grades. Almost one third of the non-users had a GPA of 4.5 or higher rather than one fifth of the users group. Two thirds of the e-cigarette users had used it at least <1 year or between 1 to 2 years, half of them used it once or twice daily and most of them used conventional cigarettes before. The most reported reasons for using e-cigarettes were reduce/quit conventional smoking because it is not a healthy habit (23%), enjoy the variability of flavors in ECs (22.3%) and economic reasons (ECs cheaper) (18%).

**Table 1:** Characteristics of participants.

Variables		E. cigarettes use		*P*-value
		Yes = 103	No = 431	
Age in years	<18	5 (4.9)	29 (6.7)	.507
	18–25 years	84 (81.6)	358 (83.1)	
	>25 years	14 (13.6)	44 (10.2)	
Gender	Male	78 (75.7)	186 (43.2)	<.001
	Female	25 (24.3)	245 (56.8)	
Year of MBBS	1st	14 (13.6)	65 (15.1)	.487
	2nd	16 (15.5)	63 (14.6)	
	3rd	30 (29.1)	88 (20.4)	
	4th	20 (19.4)	90 (20.9)	
	5th	12 (11.7)	66 (15.3)	
	6th	11 (10.7)	59 (13.7)	
GPA	<2	3 (2.9)	14 (3.2)	.001
	2.74–2.0	16 (15.5)	23 (5.3)	
	3.74–2.75	15 (14.6)	90 (20.9)	
	4.49–3.75	50 (48.5)	171 (39.7)	
	4.5 or above	19 (18.4)	133 (30.9)	
How long have you been using E. cigarettes	<1year	31 (30.1)	–	–
	1–2 years	37 (35.9)	–	
	3–4 years	26 (25.2)	–	
	5 years or more	9 (8.7)	–	
E. cigarettes per day	Occasionally (from time to time)	32 (31.1)	–	–
	Once or twice (daily base)	47 (45.6)	–	
	Regularly (your week includes some days without smoking)	24 (23.3)	–	
Why did you decide to initiate using switch to E. cigarette	Because it’s cool and fun	6 (5.8)	–	–
	Economic reasons (ECs cheaper)	19 (18.4)	–	
	Enjoy the variability of flavors in ECs	23 (22.3)	–	
	Friend or family member behaviors influence ECs use	3 (2.9)	–	
	No smoking ban in public places unlike cigarette	6 (5.8)	–	
	Normative peer pressure (the desire to be liked and belong to a group)	10 (9.7)	–	
	Reduce smoking exposure to family members.	12 (11.7)	–	
	Reduce/quit conventional smoking because it is not a healthy habit	24 (23.3)	–	
Before Electronic cigarettes did you use any of these	Cigarettes (Conventional)	74 (71.8)	–	–
	Shammah (Chewing Tobacco in the Mouth)	7 (6.8)	–	
	Shisha	22 (21.4)	–	

E. users think that e-cigarettes can be used to quit smoking as it is less dangerous than conventional cigarettes (Table 2). However, a significantly lesser proportion of E. users rather than the users, think that e-cigarettes are a source of secondhand smoking, causing various diseases, burning tobacco as a source of power and harmful to pregnant women. Furthermore, the prevalence of the 2 groups was comparable when they were asked if e-cigarette is a nicotine delivery system and it contains addictive flavor.

**Table 2:** Knowledge of E cigarettes.

Variables		E-cigarette use		*P*-value
		Yes = 103	No = 431	
Can You encourage person to quit conventional cigarette smoking for long period by using e-cigarettes	Yes	48 (46.6)	98 (22.7)	<.001
	No	55 (53.4)	333 (77.3)	
Compared to tobacco Ecs are	Absolutely harmless	7 (6.8)	21 (4.9)	<.001
	Equally harmful to tobacco cigarettes	28 (27.2)	160 (37.1)	
	Less harmful than tobacco cigarettes	48 (46.6)	105 (24.4)	
	More harmful than tobacco cigarettes	20 (19.4)	145 (33.6)	
E-cigarettes are a source of second hand smoking	Yes	58 (56.3)	322 (74.7)	<.001
	No	45 (43.7)	109 (25.3)	
E-cigarettes can cause respiratory diseases, lung, cancer, COPD or asthma	Yes	70 (68.0)	361 (83.8)	<.001
	No	33 (32.0)	70 (16.2)	
E-cigarettes is burning tobacco as sources of power	Yes	31 (30.1)	210 (48.7)	.001
	No	72 (69.9)	221 (51.3)	
E.-cigarettes is nicotine delivery system	Yes	76 (73.8)	323 (74.9)	.907
	No	27 (26.2)	108 (25.1)	
Harm effect associated more with EC use	Associated with liquid and need to be charged	33 (32.0)	154 (35.7)	.686
	Associated with the battery no liquid should be added	5 (4.9)	21 (4.9)	
	Associated with other electrical part no liquid should be added	26 (25.2)	86 (20.0)	
	Equal in the harm	39 (37.9)	170 (39.4)	
There is addictive material in the e-cigarettes with flavor	Yes	73 (70.9)	342 (79.4)	.084
	No	30 (29.1)	89 (20.6)	
Use of e-cigarettes is harmful in pregnant women	Yes	75 (72.8)	361 (83.8)	.015
	No	28 (27.2)	70 (16.2)	

Female sex is inversely associated with e-cigarette smoking (odds ratio: 0.25 (0.15–0.42), *P* < .001) (Table 3). However, age, GPA and MBBS year were not associated with E. smoking.

**Table 3:** Multivariable analysis of risk factors of E cigarettes use.

Variable		OR (95%CI)	*P*-value
Age	<18	Reference	
	18–25	0.98 (0.34–2.88)	.9
	>25	1.42 (0.41–4.90)	.58
Gender	Male	Reference	
	Female	0.25 (0.15-0.42)	**<.001**
MBBS	1st	Reference	
	2nd	1.13 (0.48–2.66)	.78
	3rd	1.30 (0.6–2.8)	.51
	4th	0.93 (0.41–2.11)	.86
	5th	0.88 (0.35–2.21)	.79
	6th	1.28 (0.51–3.25)	.6
GPA	<2	Reference	
	2.74–2.0	4.06 (0.9–18.4)	.7
	3.74–2.75	0.83 (0.19–3.6)	.81
	4.49–3.75	1.73 (0.42–7)	.44
	4.5 or above	0.91 (0.22–3.84)	.9

CI = confidence interval, GPA = grade point average, OR = odds ratio.

## 4. Discussion

In this study, we evaluated the prevalence and attitudes of e-cigarette use among 534 medical students aged 18 to 25 at King Khalid University, Aseer region, Saudi Arabia. Our study revealed that 19.3% of medical students at King Khalid University reported E-cigarette use. This rate falls between the higher prevalence of 28.2% reported at Umm AlQura University,^[25]^ and the considerably lower prevalence reported at Qassim University (10.6%).^[22]^ Meanwhile, a study conducted at King Saud University in Riyadh, where a prevalence of 25.6% among e-cigarette users was noted, reflecting the variable awareness levels among medical students.^[26]^ This discrepancy in prevalence could highlight the influence of sociocultural factors and awareness levels on e-cigarette consumption patterns.

When compared to the international setting, such as the University of Minnesota, where <1% of medical students are believed to be using e-cigarettes,^[27]^ Saudi universities, including ours, have significantly higher prevalence rates. A previous meta-analysis revealed that 17% of college students in Saudi Arabia smoke tobacco,^[24]^ surpassing the national average (12%) for individuals aged 15 to 25 by 5%. This rate exceeds those reported in neighboring regions, with college student smoking rates in Yemen,^[28]^ and the United Arab Emirates,^[29]^ noted at 12.4% and 15.1%, respectively. This extreme gap could be attributed to cultural, legal, and educational variations across the areas, as well as distinct stages in the e-cigarette adoption curve.

Our study revealed a significant gender disparity in e-cigarette usage, with males constituting 75% of users, a trend corroborated by multivariate analysis indicating a strong positive association between male gender and e-cigarette use (*P* < .001). This predominance aligns with existing literature; for instance, Alduraywish et al reported a considerable usage among first-year male university students in Saudi Arabia, with 27.4% having experimented and 13.5% actively using e-cigarettes.^[30]^

A meta-analysis indicated that male college students in Saudi Arabia smoke 21% more than their female counterparts. This gap may be related to cultural barriers, as evidenced by one study in which a male researcher encountered difficulties collecting data from female students due to social customs.^[24]^ Furthermore, social perceptions about smoking, particularly among women, may affect reporting accuracy. In the Saudi setting, smoking is frequently viewed as contrary to traditional values, which may encourage women who smoke to conceal their habits in order to escape social stigma. These findings emphasize the importance of gender-specific considerations in addressing e-cigarette use.

The rapid surge in popularity of e-cigarettes can be attributed to a number of characteristics that appeal to individuals of all ages. A combination of intense marketing methods and a lack of detailed research have positioned e-cigarettes as a preferred option for individuals who want to quit traditional smoking.^[31]^ A substantial percentage of e-cigarette users reported prior conventional cigarette usage, implying a possible transfer from traditional to electronic forms. Furthermore, our findings show that the key motivations for e-cigarette usage included a desire to quit or minimize traditional smoking, enjoyment of the various flavors available in e-cigarettes, and financial benefits, as e-cigarettes were believed to be less expensive. In terms of the factors influencing e-cigarette use, Alshanberi et al found that a minority of individuals, particularly 24.3%, perceived e-cigarettes as useful for quitting smoking.^[25]^ This outcome is consistent with the findings of our investigation, which found that 23.3% of respondents shared this view, a percentage that also matches data from Qassim University.^[22]^ In contrast, a Jeddah study found that 42.7% of e-cigarette users used these devices to help them quit smoking.^[1]^ Notably, half of those users reported successful quitting.

Recreation emerged as the leading incentive for using e-cigarettes in Jeddah study, representing 33.9% of the total usage,^[1]^ while 22.3% of individuals in our study cited the enjoyment of various flavors as their reason. This mirrors the behavior of traditional smokers, with 37.1% attributing their habit to entertainment purposes. In contrast, mental distress, which includes feelings of sadness and depression, was identified as the least prevalent reason, reported by only 4.8% of e-cigarette users. Interestingly, a regional study focusing on medical students found that a higher proportion (49%) smoked e-cigarettes for entertainment than the overall population. This study also found that medical students were slightly more likely (7.8%) to use e-cigarettes to cope with depression, which was greater than the average tendency, and 16.2% to smoke regular cigarettes for similar reasons.^[23]^ Furthermore, e-cigarettes were predominantly utilized as a method to quit traditional smoking, ranking second only to entertainment in popularity. This conclusion contrasts with findings from China, where the primary motive for using e-cigarettes was to help stop traditional smoking which align with our findings.^[32]^ In the UK, a notable 67% of respondents believed e-cigarettes to be less detrimental than traditional smoking.^[33]^ However, this view was less widespread in our study, where only 46.6% perceived e-cigarettes as less harmful, while 19.4% deemed their effects as more harmful and 27.2% considered them equally hazardous as conventional smoking. In contrast, a US study found a vast majority (84.7%) of current e-cigarette users viewing them as less harmful. This discrepancy might be linked to the educational background of the participants, predominantly high school graduates in the US survey.^[34]^ Interestingly, a separate study among medical students in Qassim University,^[22]^ reported that 69.4% viewed e-cigarettes as less harmful, though 8.2% saw them as equally dangerous as traditional smoking. This variation in perceptions among medical students across different Saudi regions warrants further exploration better understand e-cigarette use, challenges, and implications. It also encourages in-depth research of physicians’ knowledge and experiences with e-cigarettes, as well as their confidence in discussing nicotine products with patients of all ages.

The study acknowledges some limitations including the fact that population-based studies often encounter numerous confounders and biases. In our study, one significant factor is internet accessibility. Despite our best efforts, we cannot ensure the participation of the entire target population due to this limitation. Also, its dependence on self-reported questionnaires, which are susceptible to biases such as recall and selectivity, might introduce limitation. The focus on a single group limits the scope of the findings, making them less applicable to the whole Saudi population. Furthermore, the results’ validity may be influenced by a moderate response rate and the use of convenience sampling as a non-probability method. Moreover, our results included only single center of medical students; therefore, generalizability of our results is limited. Additionally, many students declined to participate as they did not understand the exact aim of our study.

## 5. Conclusion

One fifth of the medical students at King Khaled University are E. users with a large proportion of males. The health care authorities in conjunction with the educational authorities should raise the awareness level towards e-cigarettes use in particular the reasons for usage as well as it is harmful hazards.

## Author contributions

**Conceptualization:** Nasser G. Alqahtani.

**Data curation:** Nasser G. Alqahtani, Amr Ehab El-Qushayr.

**Formal analysis:** Nasser G. Alqahtani, Amr Ehab El-Qushayr.

**Investigation:** Nasser G. Alqahtani.

**Methodology:** Nasser G. Alqahtani, Reem Hamad S. Aldosari, Lama Gasem Asiri, Lujain Y. AlKasi, Shatha A. Almasswary, Alshaima Alassim, Aljohrah M. Al Hunaif, Shuruq Abdullah M. Alqahtani, Miad A. Abu Mughaedh, Amr Ehab El-Qushayr.

**Project administration:** Nasser G. Alqahtani.

**Resources:** Reem Hamad S. Aldosari, Lama Gasem Asiri, Lujain Y. AlKasi, Shatha A. Almasswary, Alshaima Alassim, Aljohrah M. Al Hunaif, Shuruq Abdullah M. Alqahtani, Miad A. Abu Mughaedh.

**Software:** Amr Ehab El-Qushayr.

**Supervision:** Amr Ehab El-Qushayr.

**Validation:** Nasser G. Alqahtani.

**Visualization:** Nasser G. Alqahtani.

**Writing – original draft:** Nasser G. Alqahtani, Amira Mohamed Taha, Reem Hamad S. Aldosari, Lama Gasem Asiri, Lujain Y. AlKasi, Shatha A. Almasswary, Alshaima Alassim, Aljohrah M. Al Hunaif, Shuruq Abdullah M. Alqahtani, Miad A. Abu Mughaedh, Amr Ehab El-Qushayr.

**Writing – review & editing:** Nasser G. Alqahtani, Amira Mohamed Taha, Reem Hamad S. Aldosari, Lama Gasem Asiri, Lujain Y. AlKasi, Shatha A. Almasswary, Alshaima Alassim, Aljohrah M. Al Hunaif, Shuruq Abdullah M. Alqahtani, Miad A. Abu Mughaedh, Amr Ehab El-Qushayr.
